# Impact of collective orientation on the quality of teamwork of emergency medical personnel in simulated prehospital emergency medical care - a prospective observational study

**DOI:** 10.1186/s12873-025-01399-2

**Published:** 2025-11-07

**Authors:** Lennart Meyer, Hendrik Eismann, Gordon Heringshausen, Vera Hagemann, Jan Carlo Del Tedesco, Markus Flentje

**Affiliations:** 1 Rescue Service Cooperation in Schleswig-Holstein (RKiSH), RKiSH Akademie, Esmarchstr. 50, 25746 Heide, Germany; 2 Department of Anaesthesiology and Intensive Care Medicine, Hannover Medical School, Carl-Neuberg-Str. 1, 30625 Hannover, Germany; 3 Akkon University of Human Sciences, Colditzstr. 34 – 36, 12099 Berlin, Germany; 4 Faculty of Business Studies and Economics, Business Psychology, University of Bremen, Enrique-Schmidt-Strasse 1, 28359 Bremen, Germany

**Keywords:** Collective orientation, Non-technical skills, Teamwork, Emergency medical personnel, Quality of care, Simulation

## Abstract

**Background:**

High-responsibility teams in prehospital emergency medicine regularly face multidimensional challenges. Collaboration in such teams is vital for the care, safety and outcome of our patients. Collective Orientation (CO) is simply defined as the individual willingness to work with others in a team. Collective Orientation has been proven to have a positive effect on team performance in clinical healthcare. However, data on prehospital emergency medicine is still lacking.

**Method:**

This prospective observational study measured the collective orientation of *n* = 64 paramedic students in their third year of training using a validated questionnaire. Teams were formed based on the students’ collective orientation (high or low). Two simulation scenarios (tension pneumothorax and bronchospasm) were evaluated using the Time Key Item Product (TKIP) and Team Emergency Assessment Measure (TEAM).

**Results:**

There was no significant difference in the speed and completeness of patient care (TKIP) between low (M = 6605) and high (M = 6967) Collective Orientation (*p* = 0.133). There was no significant difference in time to diagnosis between low (M = 384 s) and high (M = 405 s) CO (*p* = 0.128). Teams with high CO undoubtedly achieved better results in teamwork-relevant factors. These factors include leadership (*p* = 0,031), teamwork (*p* = 0,052) and task management (*p* = 0,038). There was no significant difference between low and high CO when using cognitive aids (*p* = 0,471) or the 10-for-10 principle (*p* = 0,735).

**Conclusion:**

Collective Orientation must be considered a component of paramedics’ competence to act in paramedic education, as it is characteristic of good teamwork. Further research is needed. The study suggests that Collective Orientation only works in highly complex emergencies outside of linear standard operating procedures or when combined with unanticipated complications. It is clear that leading symptoms that require a wide differential diagnosis demonstrate the value of a high collective orientation.

**Supplementary Information:**

The online version contains supplementary material available at 10.1186/s12873-025-01399-2.

## Background

Prehospital emergency medicine is characterised by emergency personnel working within interprofessional teams that are in a constant state of change. This form of teamwork is characteristic of High Responsibility Teams (HRT), and is analogous to those found in aviation, nuclear energy, law enforcement and the fire service. In this context, the field of emergency medicine is understood as a High Reliability Organization (HRO). This is due to the high level of responsibility required of personnel and the reliability of operational work processes. In the absence of this, incidents and errors of various causes would have serious consequences for patients without the trust of the teams involved [1].

The impact of effective communication and teamwork on mortality and morbidity in patient care has been demonstrated in numerous studies [2, 3]. It is evident that teamwork plays a pivotal role in reducing errors, thereby ensuring optimal patient safety. It has been demonstrated that positive effects can also be observed in relation to the adherence of care to guidelines and the prevention of infections [3]. The characteristics of management and leadership, solidarity within the team, mutual trust, team cohesion and shared mental models have been demonstrated to significantly strengthen the collective feeling and team performance [4].

The willingness of all individuals to collaborate in a team is of particular importance in successfully overcoming challenges in HRT. Driskell et al. describe this propensity to work in a team together as Collective Orientation (CO) [5]. Individuals with a high CO tend to exhibit goal-oriented and cooperative behavioral patterns, demonstrate effective collaboration, actively seek input, and make a significant contribution to the overall outcome. They also tend to associate teamwork with positive emotions. The measurement of CO is achieved through the utilisation of a standardised questionnaire, encompassing the sub-scales entitled “Affiliation” and “Dominance” [6]. Training has been demonstrated as a means to effect change in the individual CO [7]. A study conducted in a non-medical setting has demonstrated that the carbon monoxide (CO) levels of the team leader are also deemed pertinent to the team’s processes. Elevated CO levels within a team have been observed to enhance team performance [8, 9]. It has been demonstrated in previous studies that carbon monoxide (CO) exerts a positive effect, as evidenced by the findings of a pre-post study conducted by the present researchers. The present study investigated the effects of crew resource management team training on the CO of the participants [10]. Irrespective of professional group, gender and degree of engagement in the simulation component of the courses, the training program has been demonstrated to engender an increased sense of affiliation [11]. The variables of this effect are as yet unknown, as is the impact it will have on emergency medical services [10, 12].

The objective of this study was to evaluate the impact of individual paramedic students’ inclination towards teamwork on the quality of simulated prehospital emergency care. The results will be used to confirm whether CO is an effective characteristic of prehospital teamwork, and to integrate it into an educational curriculum. The hypotheses were as follows: Paramedic teams with elevated levels of CO have been shown to provide patients with faster and more comprehensive care (a). It has been demonstrated that paramedic teams with elevated levels of CO are able to communicate a diagnosis with greater expediency (b). The CO has been demonstrated to correlate with the teamwork observed in scenario (c). Paramedics utilising high CO levels have been observed to employ cognitive aids and crew resource management tools with greater frequency (d). In the future, the characteristic of CO can serve as a target and evaluation parameter in the professional training of paramedics (e).

## Methods

### Study design

**Fig. 1:**
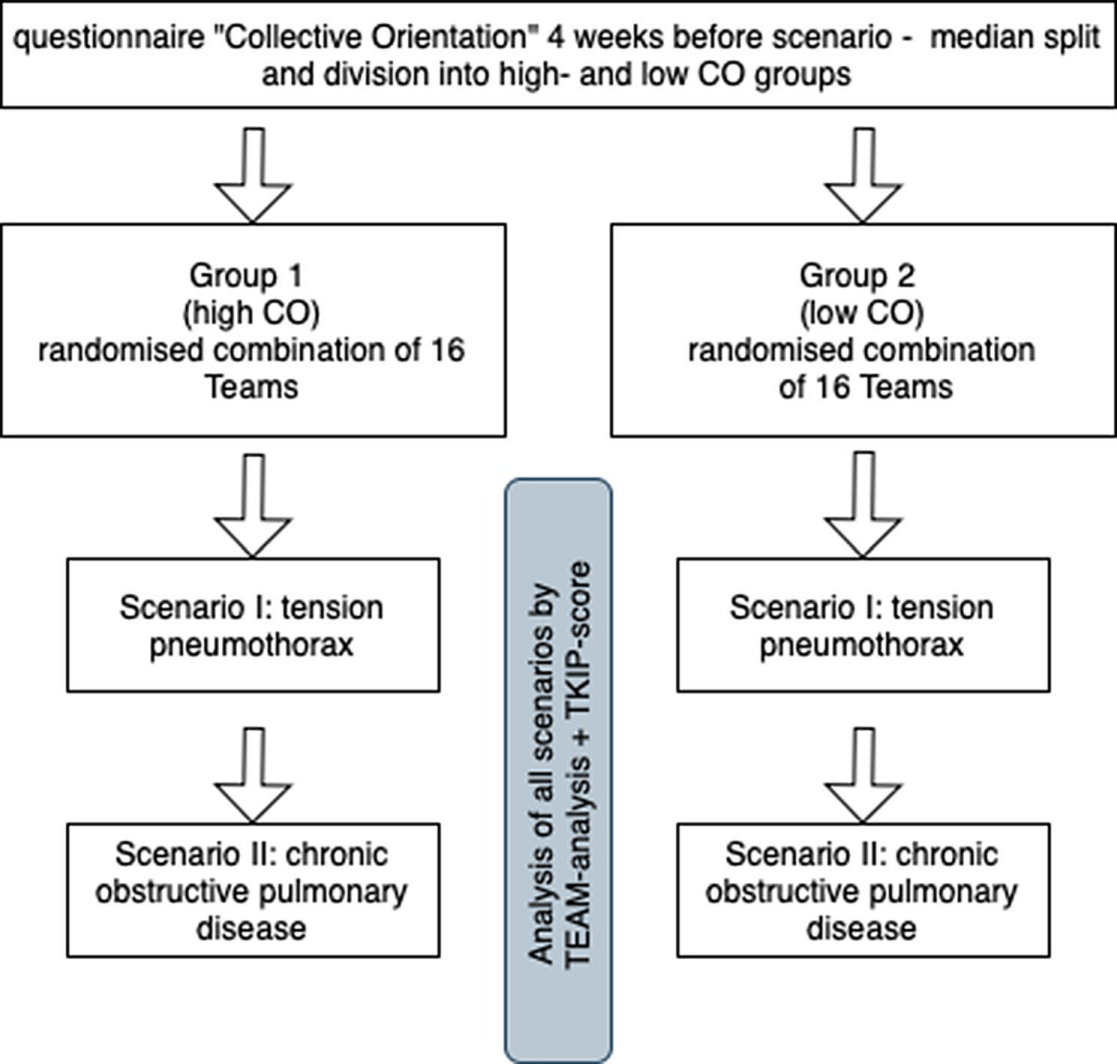
Study design. After dividing the teams into high- and low collective orientation groups, all scenarios were evaluated using the TKIP-score and the TEAM-analysis (CO: Collective Orientation)

The study was designed as a prospective observational study in a monocentric cross-sectional design. Following the measurement of collective orientation (CO) using a validated questionnaire, the participants completed two teamwork simulation scenarios on the leading symptom of dyspnea. Various technical and non-technical teamwork items were analysed in the subsequent analysis. The present study examined the effects of teamwork on the quality of simulated prehospital emergency care. The flowchart is illustrated in Fig. 1. The ethics committee of Hannover Medical School (no. 11111_BO_K_2023) approved the study, issuing an advisory result that was without reservations.

### Setting and population

The study was conducted with paramedic students of the Rettungsdienst Kooperation in Schleswig-Holstein gGmbH in a multi-stage procedure. The number of cases was set at a minimum of *n* = 32 teams (16 teams with low CO, 16 teams with high CO), consisting of two people, as indicated by other studies [10, 14]. In addition to the voluntary participation in the survey, the inclusion criterion was defined as being a paramedic student in the third year of training and thus immediately before the final state examination.

In order to facilitate subsequent evaluation and planning of the simulation scenarios, the participants were required to use a pseudonym in the online survey. The results of the questionnaire on the characteristics of CO were then used to form two groups: low and high CO. These groups were created through the implementation of a median split. Within these groups, the teams were randomly assigned pseudonyms, and subsequently randomized.

The scenarios were presented in a realistic setting using a high-fidelity simulator (SimMan 3G, Laerdal Medical, Stavanger, Norway) in a simulation environment with which the trainees at the RKiSH Academy were already familiar. The deployment of standard emergency medical equipment was requisite.

Following a preliminary briefing, each team performed the tension pneumothorax scenario, followed immediately by the bronchospasm scenario. The scenario duration was predetermined as 10 min per scenario, communicated to the test subjects, and the scenario was terminated once the stipulated time had elapsed. The criteria for clinical performance in the scenarios can be found in the supplementary material 3.

### Collective orientation

CO levels were measured using a questionnaire that had been validated in the German language [13]. The questionnaire under consideration comprises 16 items, which are divided into two subscales: Affiliation (10 items) and Dominance (6 items). Respondents are invited to indicate their level of agreement with each item using a five-point Likert scale (Cronbach’s alpha of ≥ 0.74, reliability is described as acceptable to good) [13]. The administration of the questionnaire was conducted using the web-based application SoSciSurvey (Version 2.0, SoSci Survey GmbH, München, Germany).

### Data collection

The time key item product (TKIP) is a tool employed for the evaluation of the quality of care in simulation scenarios [14]. The objective of the collection was to measure performance and evaluate the scenarios. It is therefore evident that pivotal elements of medical care are predetermined and subsequently considered during the evaluation process. The subsequent calculation of the remaining scenario time will allow conclusions about the performance to be drawn. Drawing upon extant research findings and the standard operating procedures of the RKiSH, essential technical skills (key items) of prehospital care were coded in two criteria catalogues for tension pneumothorax and bronchospasm. The Mangold INTERACT^®^ videography software (Version CM 3-3208532, Mangold International, Arnstorf, Germany) was utilised to analyse the video recordings and document the time at which a previously defined intervention was completed. In addition to the time of execution, the remaining time until the conclusion of the scenario (10 min = 600 s) is of particular importance in this evaluation. It was demonstrated that the higher the remaining time and thus the value, the faster the indicated intervention was carried out in terms of patient care and the better the performance. In the event that an intervention was only carried out incompletely or not at all by the conclusion of the simulation scenario, the remaining time of the item was given as 0 s. Furthermore, the items entitled “Use of standard operating procedures” and “Performing a 10-for-10” (team-timeout in emergency medicine – 10-for-10: 10 s to analyse the actual situation with planning of the next 10 min) were recorded in the video evaluation, but were not utilised in the TKIP, as these are non-technical skills.

The raw data was processed using Microsoft Excel (Version 16.16.27, Microsoft Corporation, Redmond, USA). Following the aggregation of the COs of the teams (low and high), the individual items of the TKIP ratings were listed with regard to execution, time, and the calculated remaining time up to 600 s. The TKIP value of the scenario was then calculated, and the total number of items fulfilled was determined. The time taken to complete more than 50% (≥ 6 items) and more than 75% (≥ 8 items) of the required items was also recorded.

In order to evaluate non-technical competencies and collaborative performance, the Team Emergency Assessment Measure (TEAM) was incorporated into the study population. The TEAM score, which has been validated, comprises 11 items, which are distributed across three subscales: leadership (2 items), teamwork (7 items), and task management (2 items). These items are subjectively coded by raters using a five-point Likert scale. Finally, the overall performance of the team is evaluated using a global rating scale (GRS) ranging from 1 to 10 [15, 16]. In the present observational study, the scenarios were evaluated by two independent raters, who conducted their assessments in a double-blind manner.

### Statistical analysis

The statistical analysis was calculated using IBM SPSS Statistics (Version 29.0.1.0, IBM, Armonk, USA). Following the collection of the relevant data, a Kolmogorov-Smirnov test was conducted for normal distribution. In the context of normally distributed data, the mean values and standard deviations were determined through exploratory data analysis. Subsequent to this, statistical differences were analysed using a t-test for independent samples. For non-normally distributed data, the median and interquartile range (IRQ) were calculated, and statistical differences were identified using the Mann-Whitney U test. The statistical significance of the results was defined as *p* < 0.05. The results of the Time to Completion 50% and Time to Completion 75% were presented as a Kaplan-Meier curve, with the objective of visualising the Time to Completion of the items in the different characteristic values and simulation scenarios [17]. The presence of any discrepancies between the Kaplan-Meier curves was ascertained through the implementation of a log-rank test (including a chi-square test). Following the assessment of interrater reliability (see supplementary material 4), the evaluation of the TEAM score incorporated the aggregation of ratings across the three subscales and the GRS from two raters, encompassing the two scenarios. The maximum scores are as follows: leadership: 32, Collaboration: 112, task management. 32, and GRS: 40. As previously outlined, statistical differences were identified in the comparison of scale values.

## Results

A total of 70 paramedic students completed the online questionnaire. During the course of the study, six participants had to withdraw due to illness; nevertheless, the minimum of *n* = 32 teams, i.e. 64 students, was still met.

### Collective orientation

For students in the third year of training (*n* = 50), the median CO was 3.31 (interquartile range (IQR) 0.56). In the second year of training (*n* = 20), the median was found to be 2.97 (interquartile range (IQR) 0.75). Paramedic students in their second year did not fulfil the necessary inclusion criteria; however, it was imperative that the requisite number of teams was reached. Second year students were only allowed to participate as team members, but not as team leaders. The paramedic students in both years were then divided into two groups, high and low CO, by means of a median split.

### Quality of care and team behavior

The quality of care was measured using TKIP and “Time to Diagnosis” scores. The results of the study are presented in Table 1. The results obtained from the study vary depending on the level of CO present. While teams with elevated CO levels have been shown to achieve superior TKIP values, those with low CO levels have been observed to attain a diagnosis more expeditiously. The findings of this study are not deemed to be of significant consequence. The results of the team ratings are displayed in Table 2. The TEAM Score demonstrates a discrepancy between the teams in favour of the high CO. The subscales of leadership and task management have been found to be significant. The correlation between CO and the number of CRM skills performed is illustrated in Table 3.

**Table 1:** TKIP and time to diagnosis of the simulated scenarios

	Tension pneumothorax			Bronchospasm			Total of both scenarios		
	Low CO	High CO	*p*	Low CO	High CO	*p*	Low CO	High CO	*p*
TKIP	3168 (IQR 721)	3380.5 (IQR 1160)	0,111 **ns**	3431,33 (IQR 754)	3592,66 (IQR 408)	0,517 **ns**	6605 (IQR 776)	6967 (IQR 1266)	0,133 **ns**
Time to diagnosis (s)	182 (IQR 103)	219,5 (IQR 191)	0,830 **ns**	169 (IQR 103)	205 (IQR 60)	0,477 **ns**	384 (IQR 185)	405 (IQR 222)	0,128 **ns**

Data is shown as median and interquartile range (IQR), s: seconds, CO: collective orientation, ns: not significant

**Table 2:** Team emergency assessment measure of both scenarios

TEAM score	Low CO	High CO	*p*
Leadership	24 (IQR 4)	27 (IQR 6)	0,031 **s**
Teamwork	88,5 (IQR 9)	94,5 (IQR 12)	0,052 **ns**
Task Management	24 (IQR 4)	26,5 (IQR 5)	0,038 **s**
Global Rating Scala	28,5 (IQR 5)	33,5 (IQR 5)	0,063 **ns**

Data is shown as median and interquartile range (IQR), CO: collective orientation, ns: not significant, s: significant

**Table 3:** Impact of CO on CRM skills “Use of standard operating procedure” and “Performing a 10-for-10”

		Tension pneumothorax			Bronchospasm		
		Low CO	High CO	*p*	Low CO	High CO	*p*
Use of SOP	Frequency	*n* = 5 (31,3%)	*n* = 3 (18,8%)	0,685 **ns**	*n* = 14 (87,5%)	*n* = 15 (93,8%)	1,000 **ns**
	Point of time (s)	283	229	0,471 **ns**	273	248	0,163 **ns**
Performing 10-for-10	Frequency	*n* = 8 (50%)	*n* = 9 (56,3%)	0,723 **ns**	*n* = 3 (18,8%)	*n* = 8 (50%)	0,063 **ns**
	Point of time (s)	383,5	425	0,735 **ns**	477	381	0,524 **ns**

Data is shown as median, SOP: standard operating procedure, s: seconds, CO: collective orientation, ns: not significant

### Time to completion 50% and 75%

Furthermore, an examination was conducted of the Time to Completion (TTC) exceeding 50% (six items) and 75% (eight items) of the defined key items in the TKIP of both simulation scenarios, with consideration given to the high and low CO.

**Fig. 2:**
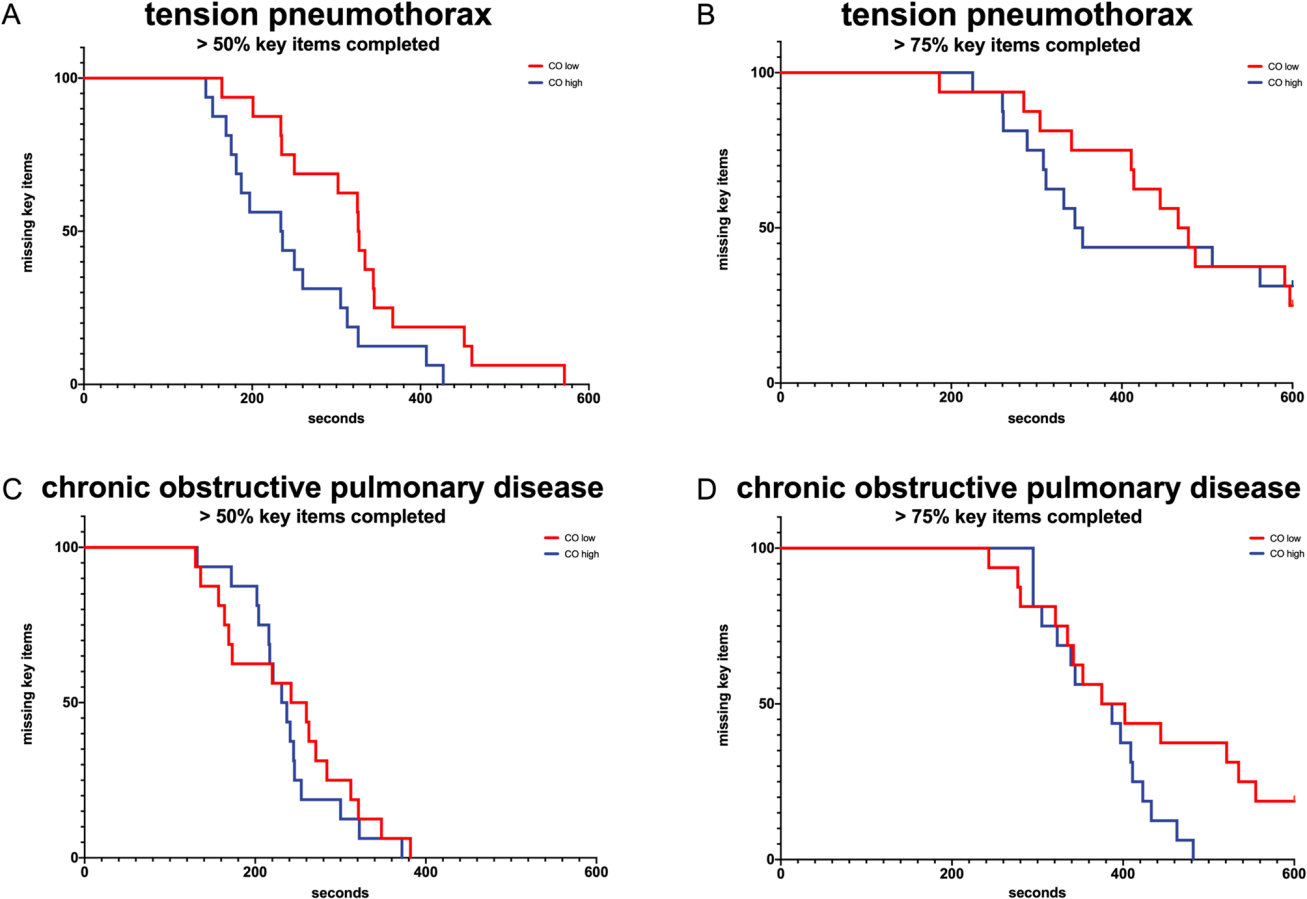
Kaplan Meyer graphs of the simulated scenarios. Key items missing for the groups “>50%” and “>75%” are depicted. Groups are shown in red (CO low) and blue (CO high). Log rank test: tension pneumothorax 50% *p* = 0,023; tension pneumothorax 75% *p* = 0,861; chronic obstructive pulmonary disease 50% *p* = 0,518; chronic obstructive pulmonary disease 75% *p* = 0,078 (CO: collective orientation)

It has been determined that in more than 50% of cases, there were differences between the tension pneumothorax and bronchospastic scenarios in terms of the time to completion. In the tension pneumothorax scenario, teams with a high CO were significantly faster to perform interventions than those with a low CO (Fig. 2A). It was observed that no specific characteristic demonstrated superiority in fulfilling 50% of the key items associated with the bronchospastic scenario (Fig. 2C).

The time to completion data demonstrated that there was no significant difference between the high and low CO groups in either the tension pneumothorax scenario or the bronchospasm scenario (see Fig. 2B and D).

## Discussion

The objective of this study was to investigate the impact of the individual Collective Orientation on teamwork of emergency medical personnel and the impact on quality of care.

The findings of the present study demonstrate that teams with a high CO do not treat patients faster and more completely than teams with a low CO. In both the tension pneumothorax simulation scenario and the bronchospasm scenario, teams with a high CO achieved better TKIP, but no difference was found. The two simulation scenarios of tension pneumothorax and bronchospasm did not involve any complications or special challenges in the study design. Consequently, the treatment of patients was facilitated through the application of standard operating procedures, a practice that was consistently adhered to by the teams. In the context of linear processing of a given standard operating procedure, the importance of cooperation within the team structure may be secondary. Whilst this finding does not support our hypothesis, it once again emphasises the importance of the utilisation of checklists and standard operating procedures in terms of patient safety, in a positive sense.

In the context of simulated patient care, emergency teams with elevated levels of CO were found to demonstrate a lack of explicit communication regarding a suspected diagnosis. In both simulation scenarios, the teams with a low CO verbalized the suspected diagnosis at a faster rate. The discrepancy in the high CO is not significant. The process of identifying a diagnosis for such high-responsibility teams may provide an explanation for this discrepancy. In the context of the study, teams with a high CO were found to have implemented a time-consuming yet broad-based differential diagnosis, predicated on leading symptoms. In contrast, teams with a low CO were found to have achieved a suspected diagnosis based on individual findings with greater expediency, albeit with reduced differentiation. This explanation can be substantiated by the frequency of the correct suspected diagnosis. In the scenario of tension pneumothorax, 75% of teams with a low CO, but 87.5% of teams with a high CO, were able to reach the correct diagnosis. The findings of this study indicate that CO is a valuable tool in circumstances where equivocal findings necessitate a comprehensive differential diagnosis, such as in cases of atypical chest pain.

A substantial discrepancy can be identified in the context of tension pneumothorax when the completion time exceeds 50%. The teams with a high CO level demonstrated a significantly faster rate of completion for essential interventions. In line with the findings that teams with a high CO took longer to diagnose, it can be hypothesised that they initiated the necessary basic interventions and symptom-oriented therapy more quickly. In the same scenario, the study revealed no statistically significant difference in the Time to Completion, with over 75% of the key items completed. In fact, it was found that not all teams achieved the required number of key items in both scenarios. For instance, the frequently omitted yet implied positioning of the patient can be hypothesised to be attributable to the ambiguous location, the diminished vigilance, a suspected generalised accident mechanism, and, within this context, the concern regarding a spinal injury. In the bronchospastic scenario, the times to completion > 50% and > 75% of the key items cannot be related to the level of CO.

Teams that achieved a high CO score demonstrated significantly superior leadership outcomes. Conversely, team leaders with a high CO have been shown to communicate instructions more effectively and maintain an overview of patient care. The findings of this study serve to reinforce the well-documented positive effects of effective leadership within prehospital high-responsibility teams, as evidenced by previous research [18, 19].

Furthermore, teams with a high CO have been observed to demonstrate a more goal-oriented and cooperative manner, as well as superior communication skills in comparison to teams with a low CO. The outcome of the team’s efforts, however, did not attain the level of significance that would be considered substantial. However, teams with a high CO demonstrate superior adherence to guidelines and prioritisation of tasks to be completed in comparison to teams with a low CO.

A high level of CO has been shown to have a positive effect on the degree of collaboration that occurs within prehospital team structures. This is evidenced by the fact that ideas from other team members are received more openly and more information is absorbed, thus enabling more rapid action to be taken in complex emergency situations. Consequently, the findings of this study support the integration of CO as a component of the professional competence of paramedics. The early initiation of the concept of collective orientation during paramedic training is of crucial importance, as is the subsequent targeted further development and monitoring of this competence in lifelong learning.

In the simulation scenarios involving tension pneumothorax and bronchospasm, no significant difference was observed between the low and high CO levels in relation to the frequency or timing of the utilisation of standard operating procedures. A discernible distinction emerges between the two scenarios: within the tension pneumothorax scenario, the utilisation of an SOP is employed by 20% to 30% of the teams, whereas in the bronchospastic scenario, the adoption of an SOP is almost universal, irrespective of the CO. The findings of the present study are of great interest and represent a significant departure from the hypothesis that was initially developed. Algorithms and checklists have been a standard component of emergency medicine for a considerable number of years. Their positive benefits have been proven, and they are also omnipresent in everyday professional life [20]. Paramedic students are made acutely aware of the significance of SOP at an early stage in their education, with a subsequent focus on the subject in their learning processes. The frequent utilisation of the medication in cases of bronchospasm can be attributed to the variety of medications available in different dosages, potential adverse drug reactions, and contraindications. Conversely, only one immediate life-saving measure is indicated for tension pneumothorax. Indeed, the two simulation scenarios demonstrate that there is no statistically significant difference in the frequency and timing of performing a 10-for-10 between the low and high CO. It is evident that the utilisation of this particular crew resource management tool is uniform across both groups, with equal frequency and concurrent usage. Notwithstanding the aforementioned similarity, 50% of teams have performed a 10-for-10 into their care. The implementation of a 10-for-10 can be evaluated in a number of ways, and it is important to adopt a nuanced and critical perspective in order to understand its implications. Paramedic students are conditioned to perform a 10-for-10 at an early stage in their education, yet if the necessary methodological competence is not acquired, i.e. at what point is a 10-for-10 appropriate, an inflationary use of the instrument can be observed, sometimes without a specific indication.

It is evident that the absence of substantial data has precluded the utilisation of CO as a universal target and evaluation parameter within the context of paramedic training. The positive effects of CO on teamwork are the only indication of this phenomenon. With regard to teamwork, the CO can be measured using the validated instrument.

The present prospective observational study has certain potential limitations. The study employed a monocentric design, with the participants being paramedic students from a substantial municipal ambulance service in one of Germany’s federal states. Whilst the presence of uniform structures, standards and equipment may appear advantageous, there is the potential for bias, thus recommending a multicentre study design.

The CO was investigated using two simulation scenarios. In this context, the simulation setting is defined as an artificial environment that is evidently distinct from actual patient care. It was imperative to disclose specific findings, as certain results were technically inadequate. It is imperative to acknowledge the observation effect in relation to the simulation. This is not only because paramedic students work differently in the simulation than in the emergency service to conform to the school’s supposed ideas and teaching statements, but also because the study population was aware of the observation in this study design. Therefore, possible observation effects could be distorted [17]. Furthermore, the two simulation scenarios were completed sequentially, which likely resulted in the teams becoming fatigued and overwhelmed by the decision-making demands.

The validated TKIP was used to evaluate performance in the two simulation scenarios. It is important to note that this instrument does not depict measures that were carried out multiple times or incorrectly. It may also be hypothesised that the TKIP method, incorporating a more extensive catalogue of criteria, could prove to be more meaningful in the context of simulation scenarios. It is evident that as the number of items in a scenario increases, there is a corresponding increase in the probability of a high CO being superior to a low CO. In consideration of the extant Standard Operating Procedures and the pertinent guidelines, it is recommended that the definition of the items in question be expanded beyond the current 11 items.

The findings indicate a necessity for further investigation into the effects of elevated CO levels. These studies will require emergency situations that are more complex and dynamic, for which there are currently no linear, often simple, standard operating procedures to demonstrate the impact of elevated CO levels. The present study hypothesises that the superiority of a high CO is demonstrated by its ability to manage unanticipated complications, such as the deterioration of the patient’s condition or adverse medication side effects, during treatment. Furthermore, symptoms that require a broad differential diagnosis can demonstrate the added value of highly pronounced CO. It is imperative that such studies encompass trained paramedics on active duty, as the experience and expertise they have acquired can have a significant impact on patient care in the context of CO. The evaluation CO in inter-professional teams comprising emergency medical technicians, paramedics and emergency physicians is also desirable.

## Conclusions

The assumption that a high CO leads to superior simulated patient care in prehospital emergency medicine is confirmed in teamwork. In the domain of emergency medical services education, CO can be regarded as a component of the non-technical competencies of prospective paramedics.

Further research is required to investigate the positive effects of CO on patient treatment. The findings indicate that the efficacy of CO is most significant in highly complex emergency situations.

## Electronic supplementary material

Below is the link to the electronic supplementary material.

Supplementary Material 1

## Data Availability

The datasets used and/or analyses during the current study are available from the corresponding author on reasonable request.

## Abbreviations

CO: Collective Orientation
CRM: Crew resource management
GRS: Global rating scale
HRO: High Reliability Organization
HRT: High Responsibility Teams
TKIP: Time Key Item Product
TEAM: Team Emergency Assessment Measure
