# Supplementary material for: Impact of collective orientation on the quality of teamwork of emergency medical personnel in simulated prehospital emergency medical care - a prospective observational study
Source: BMC Emerg Med. 2025 Nov 7;25:225. doi: 10.1186/s12873-025-01399-2 (PMC12593885; doi:10.1186/s12873-025-01399-2)
Supplement: Supplementary file 1 — Supplementary Material 1 [file 12873_2025_1399_MOESM1_ESM.docx]

**Supplementary Material**

**Material 1**: inclusion and exclusion criteria

| **Inclusion criteria** | **Exclusion criteria** |
| --- | --- |
| - paramedic students, third year - voluntary participation | - no activity in prehospital emergency medicine - no voluntary participation |

**Material 2**: simulation scenarios tension pneumothorax and bronchospasm

| scenario control | | | |  | | | | |
| --- | --- | --- | --- | --- | --- | --- | --- | --- |
| version 1.1 | | | |  |  |  |  |  |
| **tension pneumothorax** | | | | | | | | |
| Introduction to the scenario | | | | | | | | |
| **location:** | | | Apartment | | **radio call sign:** | | | 31-83-1 |
| **weather:** | | | rainy | | **safety:** | | | secured |
| **distance hospital:** | | | 20km | | **distance physician:** | | | 20km |
| **mission alert:** | | dyspnea | | | | | | |
| Mr. Kaufmann (approx. 50-year-old man) is lying on the couch. He made the emergency call himself. There is severe respiratory distress. Patient is initially somnolent. | | | | | | | | |
| Primary Survey | **A**: airway free  **B**: hypoxia  **C**: tachycardia, missing radial pulse left side  **D**: somnolent  **E**: bruise mark and crepitation left thorax | | | | | vital signs start | **consciousness**:  somnolent, protective reflexes present, pupils isocor  **breathing**:  AF 35, one-side breathing noise, SPO2 89%  **circulation**:  sinus tachycardia 160bpm, RR 80/40mmHG, ReCap 4s  **other**:  blood sugar 7,4 mmol  temp 36,4°C | |
| medical history | **S**: dyspnea, hypoxia, leck of consciousness  **A**: unknown  **M**: unknown  **P**: ladder fall one hour ago  **L**: unknown  **E**: after falling increasing dyspnea | | | | | course | **after needle decompression:**  **breathing**:  AF 20, SPO2 94%, Patient gets awake and tells about ladder fall  **circulation**:  sinus tachycardia 99bpm  RR 110/60mmHG  **without needle decompression after 7 minutes:**  GCS 8  SPO2-reduction to 60% | |

| scenario control | | | |  | | | | |
| --- | --- | --- | --- | --- | --- | --- | --- | --- |
| version 1.0 | | | |  |  |  |  |  |
| **bronchospasm** | | | | | | | | |
| Introduction to the scenario | | | | | | | | |
| **location:** | | | Apartment | | **radio call sign:** | | | 31-83-1 |
| **weather:** | | | rainy | | **safety:** | | | secured |
| **distance hospital:** | | | 20km | | **distance physician:** | | | 20km |
| **mission alert:** | | dyspnea | | | | | | |
| Mr. Kaufmann (approx. 50-year-old man) is sitting on the couch. He made the emergency call himself. He can only formulate words quietly and short of breath due to severe shortness of breath. | | | | | | | | |
| Primary Survey | **A:** airway free  **B:** expiratory wheezing, hypoxia, cyanosis  **C:** tachycardia, hypertension  **D:** awake, orientated, GCS 15  **E:** patient warm | | | | | vital signs start | **consciousness:**  awake, orientated, pupils isocor  **breathing:**  AF 28, expiratory wheezing, SPO2 89%  **circulation:**  sinus tachycardia 120bpm, RR 160/80mmHG  **other:**  blood sugar 9,1 mmol  temp 38,1°C | |
| medical history | **S:** dyspnea, expiratory wheezing, hypoxia  **A:** grasses / pollen  **M:** Formoterol spray 1-0-1  **P:** Has been treated by a doctor for 2 years with shortness of breath, nicotine abuse (10 cig/d)  **L:** breakfast 09:00 am  **E:** air has been worse for days, especially bad in the morning | | | | | course | **after inhalation of SABA & Atrovent:**  **breathing:**  AF 20, expiratory wheezing, SPO2 94%  **circulation:**  sinus tachycardia 99bpm  **without inhalation after 7 minutes:**  losing consciousness,  GCS 11  SPO2-reduction to 82% | |

**Material 3**: Key Items tension pneumothorax and bronchospasm

| scenario evaluation | |  | | | | |
| --- | --- | --- | --- | --- | --- | --- |
| version 1.0 | |  |  |  |  |  |
| **tension pneumothorax** | | | | | | |
| Key Items |  | | yes | no | Time (s) | Time remaining to 600 |
| 1 | initial assessment | |  |  |  |  |
| 2 | call out critical patient | |  |  |  |  |
| 3 | oxygen administration | |  |  |  |  |
| 4 | patient positioning | |  |  |  |  |
| 5 | auscultation of the lungs | |  |  |  |  |
| 6 | trauma examination | |  |  |  |  |
| 7 | call out diagnosis | |  |  |  |  |
| 8 | calling emergency physician | |  |  |  |  |
| 9 | check decompression criteria | |  |  |  |  |
| 10 | performing needle decompression | |  |  |  |  |
| 11 | performing a 10-for-10 | |  |  |  |  |
| 12 | load-go-and-treat decision | |  |  |  |  |
| Score |  | |  |  |  |  |

| scenario evaluation | |  | | | | |
| --- | --- | --- | --- | --- | --- | --- |
| version 1.0 | |  |  |  |  |  |
| **bronchospasm** | | | | | | |
| Key Items |  | | yes | no | Time (s) | Time remaining to 600 |
| 1 | initial assessment | |  |  |  |  |
| 2 | call out critical patient | |  |  |  |  |
| 3 | oxygen administration | |  |  |  |  |
| 4 | patient positioning | |  |  |  |  |
| 5 | auscultation of the lungs | |  |  |  |  |
| 6 | call out diagnosis | |  |  |  |  |
| 7 | 4-eyes & 6-R-rule before administration | |  |  |  |  |
| 8 | indication SABA p.i. | |  |  |  |  |
| 9 | SABA dose correct | |  |  |  |  |
| 10 | indication Prednisolon i.v. | |  |  |  |  |
| 11 | Prednisolone dosis correct | |  |  |  |  |
| 12 | performing a 10-for-10 | |  |  |  |  |
| Score |  | |  |  |  |  |

**Material 4**: Interrater reliability TEAM Score

| **subscale** | **tension pneumothorax** | | **bronchospasm** | |
| --- | --- | --- | --- | --- |
|  | **kappa** | **significance** | **kappa** | **significance** |
| leadership | 0,320 | 0,002 **s** | 0,331 | < 0,001 **s** |
| teamwork | 0,083 | 0,103 **ns** | 0,130 | 0,021 **s** |
| task management | 0,383 | < 0,001 **s** | 0,086 | 0,386 **ns** |
| global rating scale | 0,358 | < 0,001 **s** | 0,437 | < 0,001 **s** |
